# The Diseases of the Nervous System

**Published:** 1892-09

**Authors:** 


*The Diseases of the Nervous System.* By J. A. ORMEROD, M.A., M.D. Oxon. Pp. 328. London: J. & A. CHURCHILL. 1892.

Dr. Ormerod has successfully accomplished a difficult task, that of writing an account of nervous diseases which shall be short and yet omit no essential features. It seems to us that the difficulties in the way of a satisfactory book of this kind are increased by the obligation that all writers of text-books on these diseases feel, that they must include an account of the anatomy of the nervous system. Though we recognise the paramount importance of a thorough knowledge of this branch of anatomy as a preliminary to a sound knowledge of nervous disease, we question whether, when the space at disposal is limited, the student could not advantageously be referred to one of the excellent text-books which now exist on this subject. The author, however, doubtless fully considered this question and decided otherwise. The book as it now stands is certainly more complete for the admirable account of the subject which is given. The features in the work which strike us as especially praiseworthy are the chapter on methods of investigation, the excellent section on the methods of, and information gained from, electrical examination, and the clearness and vigour of the clinical descriptions. The latter are eminently practical and just what the beginner needs. His difficulties are anticipated and met, and he is enabled to form a clear picture of the group of symptoms which characterise both the typical case of any given disease and of the most important variations from the common type. The book shows that the author's practical experience in nervous diseases has been large, and that as a teacher he is well acquainted with the difficulties which attend their study. We regard the book as admirably adapted to meet the wants of senior students and of practitioners who wish to obtain a sound general knowledge of neurology, but who have not sufficient time or opportunity to study the more elaborate treatises on the subject, and we think that they may confidently rely upon it as one from which they will derive assistance for the needs of everyday practice. Moreover, a good introductory work such as this stimulates the desire for fuller knowledge. Many good illustrations, the great majority of which are original, add to its value. For the rest, the general arrangement of the book is good, and, with the index, adapted for ready reference; it is well printed, and we can only hope that it will meet with the success it undoubtedly deserves.

